# Clinical trial on tonal tinnitus with tailor-made notched music training

**DOI:** 10.1186/s12883-016-0558-7

**Published:** 2016-03-17

**Authors:** Alwina Stein, Robert Wunderlich, Pia Lau, Alva Engell, Andreas Wollbrink, Alex Shaykevich, Jörg-Tobias Kuhn, Heinz Holling, Claudia Rudack, Christo Pantev

**Affiliations:** Institute for Biomagnetism and Biosignalanalysis, University of Münster, Malmedyweg 15, 48149 Münster, Germany; Institute for Physiological Psychology, University of Bielefeld, Bielefeld, Germany; 2/22 Foyle Road, Bayswater, WA 6053 Australia; Institute for Psychology, University of Münster, Fliednerstraße 21, 48149 Münster, Germany; Department of ENT, University Clinic Münster, University of Münster, Cardinal-von-Galen Ring 10, 48149 Münster, Germany

**Keywords:** Tonal tinnitus, Tailor-made notched music training (TMNMT), Lateral inhibition, Clinical trial

## Abstract

**Background:**

Tinnitus is a result of hyper-activity/hyper-synchrony of auditory neurons coding the tinnitus frequency, which has developed due to synchronous mass activity owing to the lack of inhibition. We assume that removal of exactly these frequencies from a complex auditory stimulus will cause the brain to reorganize around tonotopic regions coding the tinnitus frequency through inhibition-induced plasticity. Based on this assumption, a novel treatment for tonal tinnitus - tailor-made notched music training (TMNMT) - has been introduced and was tested in this clinical trial.

**Methods:**

A randomized controlled trial in parallel group design was performed in a double-blinded manner. We included 100 participants with chronic, tonal tinnitus who listened to tailor-made notched music for two hours a day for three consecutive months. Our primary outcome measures were the Tinnitus Handicap Questionnaire and Visual Analog Scales measuring perceived tinnitus loudness, awareness, distress and handicap. Participants rated their tinnitus before and after the training as well as one month after cessation of the training.

**Results:**

While no effect was found for the primary outcome measures, tinnitus distress, as measured by the Tinnitus Questionnaire, a secondary outcome measure, developed differently in the two groups. The treatment group showed higher distress scores while the placebo group revealed lower distress scores after the training. However, this effect did not reach significance in post-hoc analysis and disappeared at follow-up measurements. At follow-up, tinnitus loudness in the treatment group was significantly reduced as compared to the control group. Post hoc analysis, accounting for low reliability scores in the Visual Analog Scales, showed a significant reduction of the overall Visual Analog Scale mean score in the treatment group even at the post measurement.

**Conclusion:**

This is the first study on TMNMT that was planned and conducted following the CONSORT statement standards for clinical trials. The current work is one more step towards a final evaluation of TMNMT. Already after three months the effect of training with tailor-made notched music is observable in the most direct rating of tinnitus perception – the tinnitus loudness, while more global measures of tinnitus distress do not show relevant changes.

**Trial registration:**

Current Controlled Trials ISRCTN04840953; Trial registration date: 17.07.2013

## Background

Tinnitus is a phantom perception that is a chronic condition in 10 to 15 % of the general population [1]. While some manage to cope with tinnitus, not everyone compensates it equally. Tinnitus is often accompanied by sleeping problems, stress and depression and can result in a significant reduction in quality of life [2]. Besides individual impairment, the economic burden caused by tinnitus is also a substantial issue [3]. Many attempts have been made to treat or even cure tinnitus, yet no treatment or intervention offers a completely satisfactory solution. This might be due to the fact that the exact neurophysiological mechanism underlying tinnitus is still not thoroughly understood.

Studies of noise-induced tinnitus have given rise to the general theory that tinnitus is triggered by injury to inner ear hair cell populations [4]. Damage to these cell populations results in reduced activation in the auditory nerve and reduced input to the auditory cortex [5]. The result is not only a loss of excitation of neurons coding the hearing loss region in the auditory cortex, but also a loss of inhibition from the primarily damaged frequency areas [6]. This triggers maladaptive plastic adjustments in the central auditory system, which culminate in maladaptive functional reorganization and alterations of spontaneous brain activity. As a result, neurons coding the frequencies at the audiometric edge become over-represented. This hyperactivity resembles the tinnitus perception caused by a lack of inhibition. While the neural generators may be primarily auditory cortical areas, non-auditory regions also play a crucial role [7–10], resulting in maladaptive attention towards tinnitus, as well as an aversive emotional response.

Many existing tinnitus treatments include specific auditory stimulation to induce changes in auditory processing that may reduce the tinnitus percept. These very heterogenic treatment strategies are subsumed under the term “sound therapies” and can differ with regard to the targeted mechanism like masking, habituation, residual inhibition, neuro-modulation or a combination of these methods [5, 6]. In the sound therapies focusing on masking, the tinnitus is continuously covered with another sound stimulus (e.g., white noise) which can distract subjects from their tinnitus [7]. Partial masking is part of the tinnitus retraining therapy [11] that is supposed to allow habituation to tinnitus. Yet, it is not clear whether there is a specific effect of the acoustic stimulation in TRT [12]. Nevertheless, supra-threshold masking can suppress tinnitus entirely in some tinnitus subjects, a phenomenon called residual inhibition [13]. However, this only lasts for seconds or minutes until the tinnitus recovers to its previous loudness. In the approaches focusing on neuro-modulation, specific sets of sounds are used to target the neural activity, which is assumed to underlie the sensation of the tinnitus. In one approach, tones with different frequencies around the tinnitus frequency are used in a specific rhythm to desynchronize tinnitus-related neuronal activity [14, 15]. However, the effectiveness of the latter remains unclear, as up to now there is no randomized clinical trial investigating this treatment strategy. So far, reviews concerning sound therapies found rather small effect sizes or no effects at all [5, 6, 8]. However, it remains unclear whether these results can be attributed to the lack of quality in research focusing on the effect of sound therapies without any additional counseling, or the small effect sizes of the treatment strategies per se [5, 8].

To contribute to the research on sound therapies against tinnitus, this study aims at evaluating the specific effect of a sound therapy focusing on induced inhibition, namely the tailor-made notched music training (TMNMT), which is described in detail below.

Inhibition, mainly lateral inhibition, can be evoked by presenting band-eliminated sounds to the auditory system [16]. The removal of a frequency band from an auditory stimulus will cause the brain to reorganize around tonotopic regions coding the frequencies within the band. Based on this assumption, a novel treatment for tonal tinnitus has been introduced and evaluated in a previous study: the tailor-made notched music training (TMNMT) [17]. During the course of 12 months, for 1 to 2 h per day, participants listened to music from which the spectral energy band of one octave around their individual tinnitus frequency had been removed. The hypothesis was that while auditory cortex neurons coding the tinnitus frequency would not get any or small afferent input, neighboring neurons would be activated and would inhibit the frequencies within the notch via lateral inhibition [16]. Okamoto et al. [17] chose the participants’ favorite music as the auditory stimulus for two reasons: music as an acoustic carrier of the treatment method has a sufficiently wide energy spectrum, including the tinnitus frequency; and the favorite music is able to increase positive attention that facilitates brain functional reorganization. TMNMT has been proved to reduce auditory evoked cortex activity measured by means of magnetoencephalography specifically for the tinnitus frequency [17]. Furthermore, behavioral change was monitored via Visual Analog Scales (VAS). Participants reported a reduction of the tinnitus loudness after the training. These effects were superior to any changes in the placebo or monitoring group after 12 months of TMNMT. After 6 month there was a reduction in loudness in the target group, however, this effect was not compared with the effect in the placebo or the monitoring group. A subsequent short-term concentrated (5 days, 6 h of daily listening) TMNMT study indicated that training was more effective in the case of tinnitus frequencies ≤ 8 kHz compared to tinnitus frequencies > 8 kHz [13]. This was partially explained by the lower musical energy content in the frequency region above 8 kHz. Furthermore, it became obvious that to obtain more stable effects, a longer period of training might be necessary [18]. However, effects of TMNMT have already been demonstrated after 3 days of training using a notch width of half an octave [19–21]. A further methodological modification of TMNMT was suggested by Stein et al. [20, 22], who investigated the role of the notch edges. By increasing spectral energy contrasts of the notch edges relative to the frequency region outside of the notch, the authors succeeded in further boosting the effect of lateral inhibition. However, with the exception of the first TMNMT study, none of the studies included a placebo group, stressing the need for further evaluation and replication. Based on the findings of those previous studies, we have integrated our knowledge in order to maximize the efficacy of TMNMT. Expanding on our previous work, the present study includes equalization of the music energy spectrum, edge enhancement and a ½ octave notch. With these potential improvements in mind, we decided to evaluate a training duration of 3 months and included a 1-month follow-up to evaluate the stability of the effects. This decision also aimed to increase applicability of the training. Furthermore, in order to include a broader population of tinnitus sufferers and to increase the sample size of the study, we loosened the study inclusion criteria compared to all previous studies allowing more hearing loss, higher age and higher tinnitus pitch frequency. TMNMT was designed to both reduce the aversive subjective perception of the tinnitus as well as lessen the general distress associated with tinnitus.

Our hypotheses were (1) a reduction of tinnitus distress measured by the Tinnitus Handicap Questionnaire (THQ) [23] and the mean score of the VAS to be superior in the treatment group compared to the placebo group and (2) a larger decrease in tinnitus related distress in the Tinnitus Questionnaire (TQ) [24], the Tinnitus Handicap Inventory (THI) [25] and each individual VAS subscale (i.e., tinnitus loudness, annoyance, handicap and distress) for the group, which received TMNMT compared to the placebo group. In order to promote transparency concerning our methods and outcome measures, we published a protocol to this study [26] in advance of the trial. The preparation, execution and reporting of this study was done in accordance with the CONSORT statement [27].

## Methods

### Trial design

**Fig. 1:**
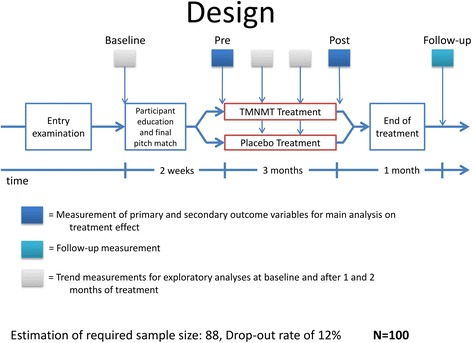
Illustration of the trial design

The clinical trial design is graphically presented in Fig. 1. This was a stratified (with balanced randomization [1:1]), double blind, placebo-controlled, parallel-group study conducted at the Institute for Biomagnetism and Biosignalanalysis of the University of Muenster, Germany. Participants were recruited through an entry examination as described herein. We invited subjects meeting the study criteria after the entry examination for the baseline measurement. We informed them about the clinical trial and instructed them how to perform tinnitus pitch matching during the following week (cf. assessment of the tinnitus frequency). On a third (pre) date, 2 weeks later, participants were finally selected for the trial. The decision was based on the outcome of the pitch-matching task. We then provided participants with either tailor-made notched music (TMNM) or placebo treatment for 3 months. Participants completed primary and secondary outcome measures at baseline, initially before treatment started (pre), at the end of the treatment (post) and at 1-month follow-up. Trend measurements consisted of the primary outcome measures.

### Participants

We recruited participants by means of advertisements in local newspapers, the homepage of the Institute, the homepage of the Faculty of Medicine of the University of Münster and by the distribution of flyers to local Ear, Nose and Throat (ENT) physicians. Potential participants were asked to answer a questionnaire covering items like the characteristics, duration, onset, possible cause, and treatment of the tinnitus as well as pre-existing illnesses, hyperacusis, current medication and drug consumption. Depending on the results of the questionnaire, potential participants were scheduled for a multidisciplinary entry investigation in the ENT department of the University Hospital of Münster. This procedure lasted approximately 2 h. It started with a screening by a psychologist, who explored the occurrence, development, and treatment of tinnitus as well as the criteria already specified in the questionnaire described above. Furthermore, tinnitus-related distress was assessed by means of standardized questionnaires (THQ, THI, and VAS). Psychological assessment was additionally supported by psychometric data using a depression scale (Allgemeine Depressionsskala – Langfassung, ADS-L) [28] and the Symptom Checklist to capture general psychological distress (SCL-90-R) [29]. After psychological assessment, an ENT physician performed a physical examination of the ear. Additionally, we measured hearing thresholds of all potential participants via an audiometric procedure using a clinical audiometer (Type Madsen Astera, Denmark) that is able to operate in an extended frequency range up to 16 kHz.

The participants recruited for the clinical trial had to meet the following inclusion criteria:

(i) Chronic (≥3 months), tonal (i.e. beep- or whistle-like) tinnitus with (ii) dominant tinnitus frequencies between 1 and 12 kHz, and (iii) without hearing loss above 70 dB HL in the frequency ranges of one half octave above and below the tinnitus frequency. In the case of bilateral tinnitus, the dominant tinnitus frequency should not differ between ears according to participants’ reports.
Aged between 18 and 70 years.
No report of severe chronic or acute mental or neurological disorders (e.g. depression, amnesia, dementia, epilepsy, etc.), which could result in major difficulties maintaining motivational compliance, the ability to follow instructions, or to participate in the training.
No acute otological diseases.
No current consumption of illegal drugs or current consumption of alcohol above the limit recommended by the World Health Organization.
No other current tinnitus therapies or other therapies that might interfere with this trial, and no participation in another clinical trial.
Written informed consent to participate in the trial.

Participants were declared as “dropouts”, if they indicated that they did not want to continue the TMNMT. If tinnitus sufferers experienced a strong increase of tinnitus loudness or started to hear an additional tone after they listened to the music for at least 8 weeks, we advised them to stop their participation in the training. Nevertheless, we asked participants who stopped performing the training to continue completing questionnaires.

The ethical aspects of this clinical trial were reviewed by an independent Institutional Review Board (Ethical Commission of the University of Münster), and ethical approval was obtained before the trial began (AZ: 2011–109-f-S). In addition, participants in the trial were fully informed about the nature, benefits, and potential dangers of the trial as well as alternative treatment options in accordance to the Good Clinical Practice Guidelines. All participants signed an informed consent form before they were enrolled in the study. They were informed that if the treatment were effective and superior to placebo, the participants of the placebo group would be provided with the target treatment after completion of the trial.

### Interventions

The intervention was conducted according to one of two protocols: (i) target (fixed notch-TMNMT) condition or (ii) placebo (moving notch) condition, described below. In both conditions, participants received a mobile device, which allowed them to perform the music training at home. Participants provided their favorite music (10 CDs), which was then imported into the music library of the mobile device and then modified in “real-time” on the device in several successive steps described below. To perform the necessary modification of the music, software for an iOS application (App) has been developed. The parameters for each condition could be entered via a graphical user interface of the App. The music was modified identically for both ears, and participants were supplied with closed headphones (model: Sennheiser HD 201).

#### Target condition

**Fig. 2:**
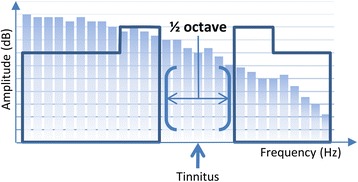
Schematic illustration of music spectrum modification (target condition). While music normally has less energy in the lower frequencies (light blue bars), TMNM is equalized. A frequency band of ½-octvae around the individual tinnitus frequency is removed from the energy spectrum of the music. The frequencies at the edge of the notch are enhanced

In the target condition, the frequency band centered at the individual tinnitus frequency of each participant was removed from the music energy spectrum in three steps: *First*, the energy spectrum of the music was “equalized”, i.e. the amplitude of the music frequency spectrum in the low and high frequency range was equalized by redistribution of energy from low to high frequency ranges. This was intended to guarantee an equal amount of spectral power below and above the frequency area suppressed by the notch filter. *Second*, a frequency band of ½ octave width centered at the individual tinnitus frequency was removed from the music energy spectrum. *Third*, the edge frequency bands of the notch were amplified using a width of 3/8 octaves on each side of the notch by 20 dB [20]. All three procedures aimed to increase the lateral inhibition effect within the notch area corresponding to the tinnitus frequency (cf. Fig. 2).

#### Control (Placebo) Condition

In the placebo condition, a moving notch filter with the same bandwidth as in the target condition was applied. The moving filter randomly selected a frequency band. After 5 s of filtering, the center frequency of the filter randomly jumped either 1/18 octave up or down and continued jumping in the same direction every 5 s until its lower or higher edge reached a predefined border at which point it changed direction.

#### Assessment of the tinnitus frequency

To achieve an appropriate estimate of the tinnitus frequency for the TMNMT, we adapted a recursive two-interval forced choice estimation of the tinnitus frequency [30, 31]. This method has been shown to produce reliable results compared to the clinical audiometry testing procedure, but it can be performed by the participants themselves using the App and in one third of the time [32]. Additionally, the procedure allowed for the analysis of the variability of an individual pitch matching session. For this procedure, the frequency range (1 to 16 kHz) was bisected into two equally large subintervals. For both the low and the high frequency subinterval, the participant had to choose whether the tinnitus was more similar to its lower or higher frequency end. Depending on the outcome, bisection and two-interval forced-choice testing was reapplied to the low subinterval, the high subinterval, or a new middle interval that was bound by the midpoints of the low and the high subinterval [30, 31]. Prior to testing, the left or right ear was selected. Loudness of the test tones was adjusted individually by participants to match the tinnitus loudness and this value was saved by the App. A two-alternative forced-choice octave confusion test completed the testing. All choices made by participants were saved in an ASCII table file. Participants performed the pitch-matching test at home twice a day for 5 days. To achieve an estimate for the tinnitus frequency as the final tinnitus frequency for the treatment, frequencies were transformed into Cent and the arithmetic mean of the ten measurements was calculated. To estimate how the values of the ten pitch measurements varied within one participant; the standard deviation was calculated for each participant.

#### Instructions for participants during TMNMT

Participants were instructed to listen to their individually modified music for two successive hours per day for 12 weeks (168 h of TMNMT in total). They were instructed to listen to the music attentively in a quiet surrounding at home with comfortable loudness. They were allowed to read, surf the internet or do other relaxing activities. Any cognitive or attention demanding task, such as working or learning, were to be avoided. Additionally, participants were instructed to avoid sound distractions by other sound sources during TMNMT.

### Outcomes

The primary efficacy endpoints in our study were at the end of the training after 3 months of music training (post) compared to pre measurement. The main outcome domains were subjective tinnitus perception and tinnitus distress.

Tinnitus perception is often measured subjectively via VAS. The reliability of those scales in tinnitus is considered as good [33], though there is a lack of studies evaluating VAS in tinnitus. To interpret the change in VAS, Adamchic, and Langguth [33] have done a psychometric evaluation, which has identified a minimally clinically important difference (MCID). To measure tinnitus related distress, a wide variety of questionnaires exists. The most common ones are the TQ, the THQ and the THI. All of those questionnaires define tinnitus handicap and distress in different ways using different theoretical concepts [34]. Besides ensuring construct validity, questionnaires as outcome measures should also be change-sensitive to detect treatment responses. Unfortunately, the authors of many tinnitus questionnaires do not provide grading systems or describe an MCID. For the TQ, a grading system exists [35], which identifies an MCID of five score points. For the THQ, an MCID of 21 score points was reported [36] and for the THI, seven score points have been suggested as an MCID [37]. Our choice of outcome measures for the current study was motivated by psychometric quality and change-sensitivity in particular. Furthermore, we wanted to meet current recommendations for questionnaires in tinnitus studies [38] and to use measures that were used in the previous TMNMT studies to achieve comparability between the studies. Therefore, we focused on THQ and VAS as primary outcome variables.

#### Primary outcome measures

(i) Tinnitus-related distress was assessed with the THQ. For statistical analyses, we used the total score.
(ii) VAS total score. Participants rated their tinnitus loudness, annoyance, awareness and handicap on separate VAS (scale from 0 to 100). The mean score from these four scales was used as the total score.

#### Secondary outcome measures

The German version of the Tinnitus Questionnaire was used as a secondary outcome measure. Furthermore, the emotional and cognitive distress subscale of the THQ and the subscales of the VAS were analyzed.

#### Additional data

The time that was spent listening to TMNM was documented on a daily basis. Participants sent the data to the study conductors via email once a week. To allow further sub-group and covariate analyses, we collected the following data: ADS-L, SCL-90-R, German Hyperacusis Questionnaire (Geräuschüberempfindlichkeits-Fragebogen, GÜF), subjective average listening duration and enjoyment of conventional music prior to study enrollment, VAS measuring different aspects of listening to TMNM during the study (enjoyment, relaxation, attention to the music), subjective experience with TMNMT (improvement of tinnitus, recommendation of TMNMT) and side effects during and after the training (open answer format). After they had completed the training, participants were asked to rate how effectively they had experienced the training on a five point Clinical Global Impression scale (CGI; cf. [28]) ranking from “very much improved” to “very much worsened”. Furthermore, they were asked to guess to which group they had belonged.

### Sample size

We performed a priori power calculations to estimate an adequate sample size to find meaningful clinical changes in our outcome variables. The calculations were performed using G*Power 3.1.5 [39]. The estimation of the expected effect size was based on an approximation rule of thumb about the minimal clinically important differences proposed by Norman, Sloan, and Wyrwich [40]. In their systematic review, they found that the threshold to determine a clinically relevant change in health-related questionnaires was approximately 0.5 standard deviations. This corresponds to a standardized effect size of d = 0.5, which can be interpreted as a medium effect [39]. The conventions for effect sizes of repeated measures ANOVAs were defined by Cohen [41] as *f* = 0.1, *f* = 0.25 and *f* = 0.4 for small, medium and large effects, respectively. Thus, we chose a medium effect size of *f* = 0.25 for our power analysis to assure that we were able to detect the assumed MCID. We planned to achieve a power of .90 for testing the interaction effect *Session* (pre, post) x *Group* (placebo, treatment) (5 % significance level). The correlation between the two repeated measures was set to *r* = 0. Note that this reflects a conservative approximation and any correlation larger than *r* = 0 would have reduced the required amount of participants. These calculations resulted in a sample size of *n* = 88. To take dropouts into account, the final sample size was set to *n* = 100, i.e. 50 participants per group.

### Interim analyses and stopping rule

In order to rule out adverse effects of the treatment, an interim analysis was conducted after 50 % of the participants completed the training (post measurement). For this purpose, data was unblinded by an external person and stored in a way that no inference to the single participant could be made (e.g. without age, gender, hearing loss, etc., only analysis relevant data). In case of a significant interaction between *Session* and *Group* concerning primary outcome measures revealing a negative effect for the treatment group, the trial would have been stopped.

### Randomization and allocation

We included 100 participants with tonal tinnitus in two study arms:

(i) Target group, 50 participants with tonal tinnitus obtaining TMNMT.
(ii) Placebo group, 50 participants with tonal tinnitus obtaining placebo treatment.

The allocation to the two groups was done using stratified randomization. Stratification is an appropriate allocation method for improving power in small trials (<400 participants) and preventing type 1 errors [42]. We used a minimum number of strata to improve statistical efficiency, since this allows the assignment of an equal number of participants to each group and assures an equal distribution of the stratification factor between groups [42]. We chose the variables age (<51 or > = 51 years) and hearing loss (<40 dB or > = 40 dB) [43, 44] as stratification categories to keep the number of strata minimal but include the two variables most likely to influence the treatment outcome. This resulted in four strata: 1) age below 51 years and less than 40 dB hearing loss, 2) age below 51 years and equal or more than 40 dB hearing loss, 3) age equal or above 51 years and less than 40 dB hearing loss, 4) age equal or above 51 years and equal or more than 40 dB hearing loss. Within each stratum, a blocked randomization (block size = 4) was performed using a computer generated randomization list. To ensure that study results were not confounded by anticipation or expectation, participants and investigators remained blind to the allocation (double blind).

### Blinding

An external person not involved in the Clinical Trial was responsible for the allocation of each participant to one of the two groups (treatment or placebo), and the App was set up accordingly by a researcher who had no contact with the corresponding participant. The allocation of a participant to the target or the placebo group was realized within the App through the application of appropriate settings. Afterwards, the App was password-locked to disable further changes and reading of the settings.

### Statistical methods

The respective hypotheses were formulated as one-sided as proposed by Owen [45]. For *p*- values which point in the opposite direction, the transformation 1-p/2 was used. To assess the effect of TMNMT, we conducted an intention-to-treat analysis with the data of all participants that were assigned to the treatment or to the placebo group. In anticipation of dropouts and missing data, we chose a likelihood-based mixed-effects model for repeated measures analysis as the primary analysis [46, 47]. The model was implemented using the function *lmer* in R’s lme4 library [48]. Here, *Subject* was set as a random effect and the factors *Session* (pre vs. post) and *Group* (treatment vs. placebo) were defined as fixed effects. Additionally, an analysis with complete cases, i.e. without dropouts (“per protocol analysis”), was conducted. This was implemented by means of a mixed model repeated measures analysis of variances (ANOVAs) for primary and secondary outcome variables with the within subject factor *Session* (pre vs. post) and the between subject factor *Group* (placebo vs. treatment). As described in the previous section, all outcome measures were assessed in a follow-up measurement 1 month after TMNMT cessation. To evaluate the time course of the effect of TMNMT, an additional mixed model ANOVA for repeated measures was conducted including the follow-up measurement. This was done with all primary and secondary outcome measures with the factors *Session* (pre vs. post vs. follow-up) and *Group* (placebo vs. treatment). If the sphericity assumption was violated in any comparison, Greenhouse-Geisser correction was applied to the data.

#### Additional analyses

For all significant results, an estimate for the effect size was calculated. Here, we used a variant of Cohen’s d, which was recommended by Morris [49] to compare effects in the treatment group to those in the control group, which is based on pooled pretest standard deviations.

To examine the influence of additional variables on the change in the primary outcome variables, we conducted several univariate analyses of covariance (ANCOVAs). As dependent variables, we used the change values of the primary outcome variables (post – pre) which were compared between the two groups. The change value was used, to facilitate the interpretation of the results. The following variables were introduced in the ANCOVA as covariates*: listening times, hearing loss, tinnitus frequency,* standard deviation of tinnitus frequency measurements for each subject *(tinnitus pitch SD)* and *age*.

Furthermore, we assessed the reliability of our two primary outcome variables by calculating the correlation coefficient between baseline and pre-training measurement. For the mean VAS value, we found a non-satisfactory low correlation between the baseline and pre-training measurement. Hence we used a latent change score analysis [50] to analyze the change in the VAS total score in more detail. In this approach, multiple-group structural equation modeling (SEM) was used to estimate a latent, error-free factor that represents group-specific change. This modeling approach is possible even in smaller samples as long as high communality is given [51].

## Results

### Participant flow

**Fig. 3:**
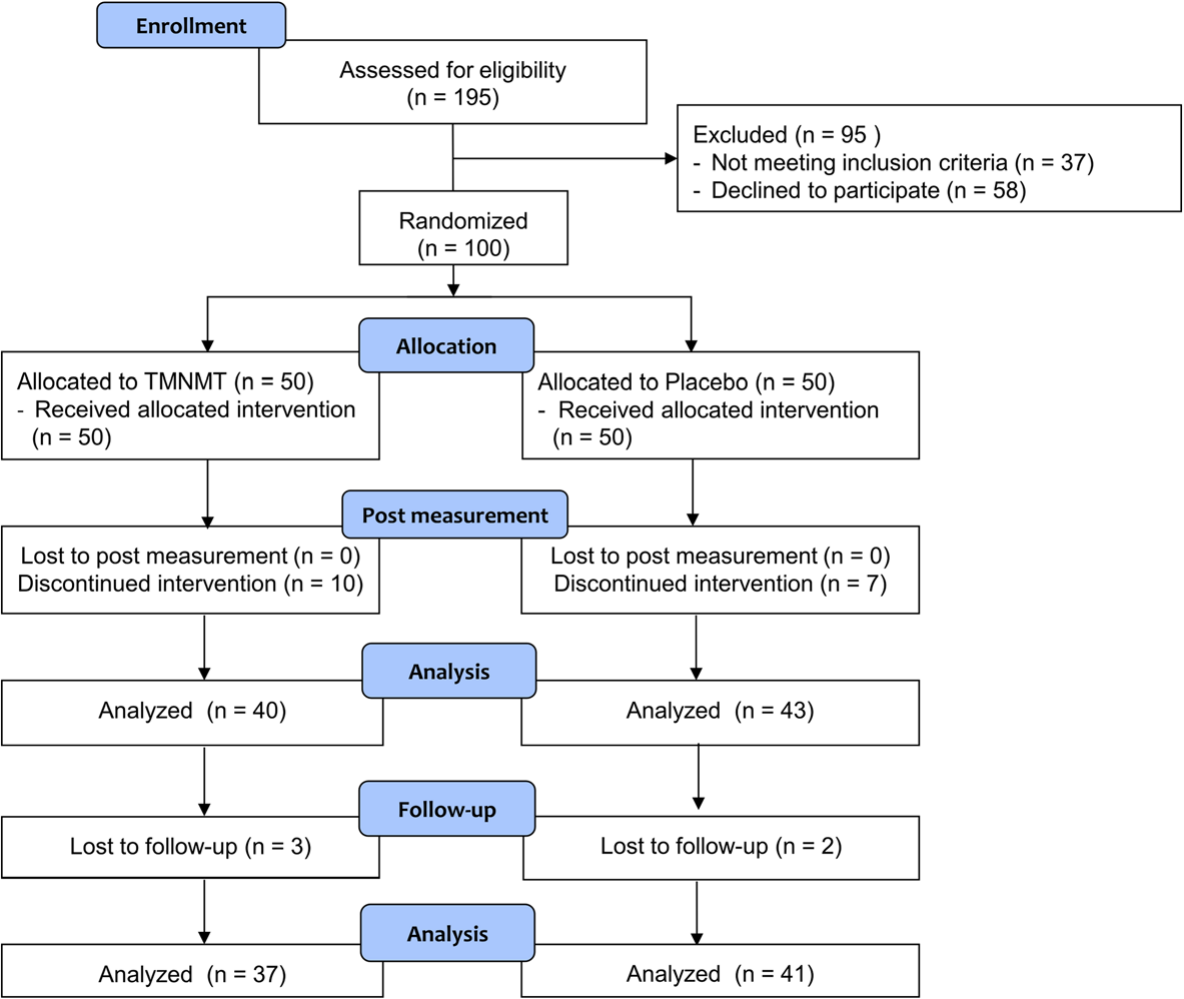
Participant Flow

An overview of the participant flow is given in Fig. 3. In total, 195 patients were screened for the study. From these participants, 37 did not meet the study criteria while 58 declined to participate in the study. The main reasons for screening failure were hearing loss (27 %) and tinnitus pitch (27 %). The main reason for participants to decline to take part in the study was a lack of time (50 %). Finally, 100 participants were included and randomly assigned to either treatment (*n* = 50) or placebo (*n* = 50) group. A total of 83 participants completed the study: 43 in the placebo group and 40 in the treatment group. The detailed numbers of analyzed participants per statistical analysis is shown in Table 1. The most frequent reasons for discontinuation in both groups were adverse effects on the tinnitus (31 %), perceived quality of the music (25 %) and that the TMNMT was experienced as too time consuming (25 %). The recruitment of the participants took place between July 2013 and June 2014, and the follow-up period of the trial was finished in October 2014. The interim analysis was conducted in May 2014 when 44 participants had finished the 3 months on TMNMT or placebo intervention. As there was no significant interaction between group and primary outcome measures revealing a negative effect for the treatment group, the trial was continued.

**Table 1:** Number of participants analyzed for each analysis individually

	Treatment	Placebo
Primary outcome measures (pre vs. post)		
THQ total score	39^a^	43
VAS total score	40	43
Secondary outcome measures (pre vs. post)		
THI	40	43
TQ	40	43
Primary outcome measures (pre vs. post vs. follow-up)		
THQ total score	37	41
VAS total score	37	41
Secondary outcome measures (pre vs. post vs. follow-up)		
THI	37	41
TQ	37	41

^a^One participant forgot to answer this questionnaire

### Baseline data

In the complete sample, the age of the participants was between 21 and 68 years (*M* = 47.5, *SD* = 10.81) with about one third (33 %) being female. The hearing loss ranged between 0 and 70 dB. 20 % of the subjects showed no clinically relevant hearing loss (<20 dB) while 20 % had a mild (20–40 dB) and 38 % a moderate (>40–70 dB) hearing loss. Table 2 shows the demographic and clinical characteristics at the baseline measurement for treatment and placebo group separately.

**Table 2:** Demographic and clinical characteristic at baseline

	Treatment	Placebo	*p*-value
	Mean (SD)	Mean (SD)	
Number	50	50	
Age (years)	47.68 (9.94)	47.13 (11.70)	.737
Gender (f/m)	17/33	16/34	.834
Hearing loss (dB)^a^	41.10 (20.26)	44.60 (21.54)	.405
Tinnitus frequency (Hz)^b^	5406 (1731)	5458 (1519)	.921
tinnitus pitch SD (Hz)^b^	1341 (1202)	1333 (1181)	.855
Time since tinnitus onset (years)	7.03 (7.25)	8.95 (6.80)	.175
THQ total score	24.95 (16.92)	28.51 (16.41)	.288
THQ emotional and cognitive distress	20.95 (19.61)	22.85 (20.20)	.635
VAS total score	42.83 (21.71)	44.71 (19.28)	.647
VAS loudness	50.70 (23.19)	48.86 (22.23)	.686
VAS handicap	28.58 (22.26)	31.36 (22.30)	.534
VAS annoyance	39.70 (26.18)	40.32 (22.27)	.899
VAS awareness	52.32 (24.58)	58.30 (21.57)	.199
THI	24.10 (14.96)	26.96 (16.92)	.373
TQ	19.12 (10,62)	25.28 (13.82)	.014
ADSL	7.80 (6.29)	9.38 (7.27)	.248
SCL-90-R	0.29 (0.28)	0.36 (0.34)	.218
GüF	8.62 (5.59)	10.58 (7.43)	.139

m = male, f = female, Hz = Hertz, dB = decibel, Tinnitus pitch SD = standard deviation of ten tinnitus pitch measurements before training

^a^maximum hearing loss in a frequency range ½ of an octave above and below the individual tinnitus frequency; ^b^Cent values retransformed into Hz to allow better interpretation

Both groups were comparable in terms of demographic characteristics and in the psychoacoustic measurements. Concerning the values in the primary and secondary outcome measures, groups did differ significantly only in the score of the TQ when all participants were included in the comparison. Note, however, that for the analysis of the secondary outcomes, we excluded the participants, who had dropped out of the intervention. Within this sample, we did not find any significant difference between the two groups in any outcome measure (all *p* > .1).

## Numbers analyzed

### Outcomes and estimations

#### Intention – to –treat analysis

The likelihood-based mixed effects model for repeated measures with *Subject* defined as the random effect and the factors *Session* (pre vs. post) and *Group* (treatment vs. placebo) as fixed effects was calculated for primary outcome measures of all participants, which took part in the study, included. There were no significant effects for both primary outcome measures (see Table 3.)

**Table 3:** Likelihood-based mixed effects model for repeated measures

	*Df*	*t*-statistic	*p*-value
THQ total score			
Session	90.14	−0.288	.774
Session x Group	90.77	−1.127	.869
VAS total score			
Session	88.52	−0.002	.999
Session x Group	88.85	0.280	.390

#### Analysis per protocol – outcomes at post-intervention

##### Primary outcome measures

The mixed model ANOVA, which was run on a sample without dropouts, with the within subjects factor *Session* (pre vs. post TMNM exposure) and the between subjects factor *Group* (treatment vs. placebo) showed neither a significant main effect for the factor *Session,* nor for the interaction effect *Group* for THQ or VAS total scores (see Table 4).

**Table 4:** Mixed model ANOVA for primary outcome measures (pre vs. post)

	*Df* (numerator, denominator)	*F*-statistic	*p*-value	*η*_*p*_^*2*^
THQ total score				
Session	1, 80	0.040	.842	.001
Session x Group	1, 80	0.678	.794	.008
VAS total score				
Session	1, 81	3.050	.085	.036
Session x Group	1, 81	1.494	.113	.018

##### Secondary outcome measures

**Fig. 4:**
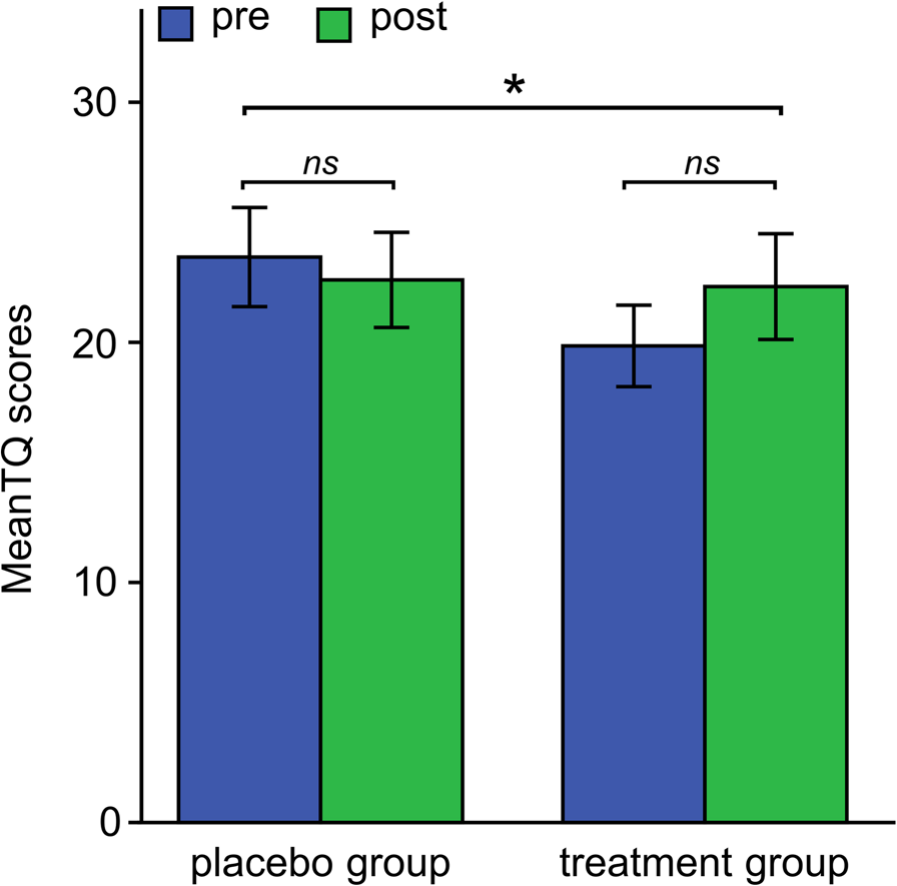
Interaction effect of Session x Group for mean TQ scores. Ns = not significant. * = *p* < .05

The mixed model ANOVA with the within subjects factor *Session* (pre vs. post) and the between subjects factor *Group* (treatment vs. placebo) showed a significant interaction effect *Session* x *Group* for the TQ, *F* (1, 81) = 4.075, *p* = .047 (two-sided), *η*_*p*_^*2*^ = .048, *d* = .182. This interaction effect revealed a reduction of TQ scores for the placebo group (pre: *M* TQ score = 23.56, *SD* = 13.56; post: *M* TQ score = 22.61, *SD* = 13.02), while TQ scores for the treatment group increased after TMNMT (pre: *M* TQ scores = 19.85, *SD* = 10.75; post: *M* = 22.33, *SD* = 13.96; see also Fig. 4). However, post-hoc analysis using paired *t*-tests did not reveal significant differences between pre and post measures for each group individually (placebo group: *t* (42) = 1.137, *p* = .262; treatment group: *t* (39) = −1.636, *p* = .110). Furthermore, a significant main effect for the factor *Session* was found for the VAS subscale awareness revealing an overall reduction of VAS ratings for both groups (pre: *M* VAS awareness ratings = 56.63, *SD* = 22.80; post: *M* VAS awareness ratings = 50.10, *SD* = 25.65), *F* (1, 81) = 8.612, *p* = .004, *η*_*p*_^*2*^ = .096. All other comparisons were not significant (see Table 5).

**Table 5:** Mixed model ANOVA for secondary outcome measures (pre vs. post)

	*df* (numerator, denominator)	*F*-statistic	*p*-value	*η*_*p*_^*2*^
THI				
Session	1, 81	0.076	.783	.001
Session x Group	1, 81	0.049	.588	.001
TQ				
Session	1, 81	0.803	.373	.010
Session x Group	1, 81	4.075	.977*	.048
THQ factor 1				
Session	1, 80	1.009	.318	.012
Session x Group	1, 80	0.973	.837	.012
VAS loudness				
Session	1, 81	2.293	.134	.028
Session x Group	1, 81	2.369	.064	.028
VAS annoyance				
Session	1, 81	0.441	.508	.005
Session x Group	1, 81	0.741	.196	.009
VAS awareness				
Session	1, 81	8.612	.004***	.096
Session x Group	1, 81	0.288	.297	.004
VAS handicapping				
Session	1, 81	0.120	.730	.001
Session x Group	1, 81	1.211	.137	.015

*Note, that this *p*-value would be considered as significant under a two sided testing (*p* = .047*)

*** = *p* < .01

#### Analysis per protocol – outcomes at follow-up

##### Primary outcome measures

We calculated an additional mixed model ANOVA with the within subjects factor *Session* (pre vs. post vs. follow-up) and the between subjects factor *Group* (treatment vs. placebo). Again, we found no significant effects for the primary outcome measures THQ and VAS total score (see Table 6). Since no hypotheses for follow-up measurements were formulated in the protocol, the respective *p*-values are reported as two-sided.

**Table 6:** Mixed model ANOVA for primary outcome measures (pre vs. post vs. follow-up)

Effect	*df* (numerator, denominator)	*F*-statistic	*p*-value	*η*_*p*_^*2*^
THQ total score				
Session	1.72, 130.97^a^	0.666	.494	.009
Session x Group	1.72, 130.97^a^	1.183	.305	.023
VAS total score				
Session	1.42, 108.24^a^	2.969	.073	.038
Session x Group	1.42, 108.24^a^	1.781	.182	.023

^a^Greenhouse-Geisser corrected

##### Secondary outcome measures

**Fig. 5:**
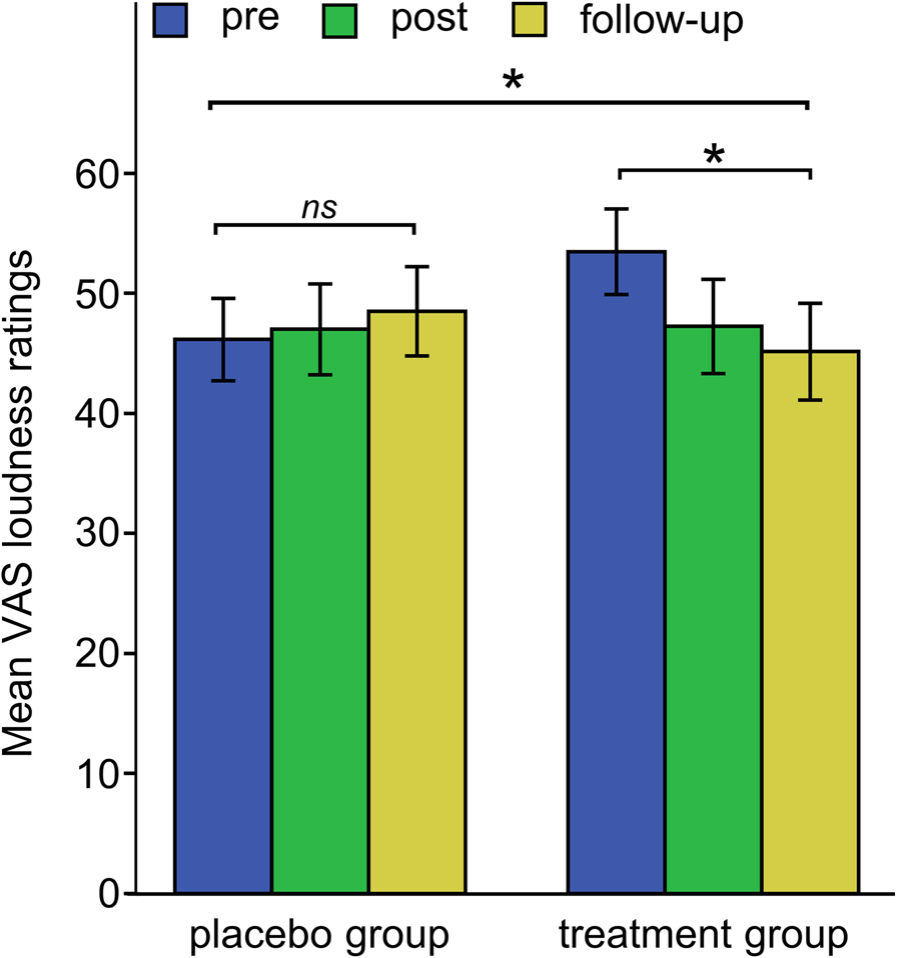
Interaction effect of Session x Group for mean VAS loudness ratings. ns = not significant. * = *p* < .05

For the secondary outcome measures, we found a significant interaction effect *Session* x *Group* for VAS loudness ratings, *F* (1.64, 124.95) = 4.262, *p* = .022, *η*_*p*_^*2*^ = .053, d = .266, revealing a reduction of tinnitus loudness in the treatment group (see Fig. 5). We calculated post-hoc tests using repeated measures ANOVA with the between subjects factor *Session* (pre, post, follow-up) for each group individually which revealed a reduction of tinnitus loudness in the treatment group (pre: *M* VAS loudness ratings = 53.46, *SD* = 21.74; post: *M* VAS loudness ratings = 47.24, *SD* = 23.80; follow-up: *M* VAS loudness ratings = 45.14, *SD* = 24.45), *F* (1.58, 56.87) = 4.014, *p* = .032, *η*_*p*_^*2*^ = .10; while there was no main effect of *Session* for the control group (pre: *M* VAS loudness ratings = 46.15, *SD* = 21.89; post: *M* VAS loudness ratings = 47.00, *SD* = 24.16; follow-up: *M* VAS loudness ratings = 48.49, *SD* = 23.86), *F* (1.69, 67.76) = 0.576, *p* = .537, *η*_*p*_^*2*^ = .01. Furthermore, there was a significant main effect *Session* for VAS awareness ratings, *F* (1.56, 118.86) = 6.579, *p* = .004, *η*_*p*_^*2*^ = .080, revealing an overall decrease of tinnitus awareness ratings after TMNM exposure (pre: *M* VAS awareness ratings = 56.74, SD = 22.84; post: *M* VAS awareness ratings = 50.10, *SD* = 26.00; follow-up: *M* VAS awareness ratings = 49.62, *SD* = 25.65). All other comparisons were again non-significant (see Table 7).

**Table 7:** Mixed model ANOVA for secondary outcome measures (pre vs. post vs. follow-up)

Effect	*df* (numerator, denominator)	*F*-statistic	*p*-value	*η*_*p*_^*2*^
THI				
Session	1.76, 133.98^a^	0.113	.870	.001
Session x Group	1.76, 133.98^a^	0.074	.908	.001
TQ				
Session	1.57, 119.30^a^	0.758	.442	.010
Session x Group	1.57, 119.30^a^	2.459	.102	.031
THQ factor 1				
Session	1.66, 126.37^a^	2.335	.110	.030
Session x Group	1.66, 126.37^a^	0.332	.678	.004
VAS loudness				
Session	1.64, 124.95^a^	1.567	.215	.020
Session x Group	1.64, 124.95^a^	4.262	.022*	.053
VAS annoyance				
Session	1.51, 114.90^a^	1.205	.294	.016
Session x Group	1.51, 114.90^a^	1.440	.241	.019
VAS awareness				
Session	1.56, 118.86^a^	6.579	.004**	.080
Session x Group	1.56, 118.86^a^	0.041	.928	.001
VAS handicapping				
Session	1.56, 118.71^a^	0.263	.714	.003
Session x Group	1.56, 118.71^a^	1.321	.267	.017

^a^Greenhouse-Geisser corrected

* = *p* < .05; ** = *p* < .01

### Ancillary analyses

#### Covariate analyses

To exclude possible influences of third variables, we calculated univariate ANCOVAs for the difference values (post – pre TMNM exposure) of the primary outcome measures. Here, we found a significant covariance between listening times total and VAS total change values, *F* (1, 68) = 2.036, *p* = .019, *η*_*p*_^*2*^ = .079. However, treatment and placebo group did not differ in VAS total change values after correction for the influence of the factor listening times total, *F* (1, 68) = 1.666, *p* = .201. No other comparisons showed significant effects, as described in Table 8.

**Table 8:** Mixed model ANCOVA for difference values (pre vs. post) of primary outcomes

	*df* (numerator, denominator)	*F*-statistic	*p*-value	*η*_*p*_^*2*^
Change VAS total				
Hearing loss (HL_1/2oct)^a^	1, 80	0.944	.334	.012
Group (HL_1/2oct corrected)	1, 80	1.594	.217	.019
Listening times total (LTT)	1, 68	2.036	.019*	.079
Group (LTT corrected)	1, 68	1.666	.201	.024
Tinnitus pitch SD^b^	1, 80	3.839	.054	.046
Group (tinnitus pitch SD corrected)	1, 80	1.406	.239	.017
Age	1, 80	0.167	.684	.002
Group (Age corrected)	1, 80	1.534	.219	.019
Change THQ total				
Hearing loss (HL_1/2oct)	1, 79	1.196	.277	.015
Group (HL_1/2oct corrected)	1, 79	0.621	.433	.008
Listening times total (LTT)	1, 67	0.244	.623	.004
Group (LTT corrected)	1, 67	0.157	.693	.002
Tinnitus pitch SD^b^	1, 79	1.633	.205	.020
Group (tinnitus pitch SD corrected)	1, 79	0.752	.389	.009
Age	1, 79	.500	.481	.006
Group (Age corrected)	1, 79	.592	.444	.007

^a^HL_1/2oct = hearing loss in the range of ½ octave around the individual tinnitus frequency; ^b^Tinnitus pitch SD = standard deviation of ten tinnitus pitch measurements before training

* = *p* < .05

#### Structural Equation Model for VAS total values

Since VAS scales showed a low test-retest reliability between both measurements before TMNM exposure (*r* = .67), we performed a latent change score analysis. In this approach, a SEM for both treatment and control groups with a number of constraints was specified (see [50], Fig. 3 for a similar model). First, the model included a pre-intervention and a post-intervention factor based on the 4 VAS items investigated here. The constraints with respect to these factors were that (a) all factor loadings had to be identical for pre-intervention and post-intervention factors as well as (b) for both groups, (c) all residual correlations between pre-intervention and post-intervention items were equal between groups, and (d) the path from pre-intervention to post-intervention factor was fixed to 1 for identification purposes [52]. Furthermore, a latent change factor was included, representing the difference between pre and post. This factor was identified by constraining its path to the post-intervention factor to 1. Finally, latent means of the pre-intervention and post-intervention factors were fixed to 0, and the mean of the change factor was fixed to 0 in the control group.

Overall, model fit was good, *χ*^*2*^(60) = 76.62, *p* > .05, CFI = .97. Critically, the mean of the latent change factor in the treatment group differed significantly from 0, standardized *α* = .238, *p* = .034 (one-sided). This result implies that the VAS change in the treatment group was significantly larger than in the control group. The standardized alpha may be interpreted similarly to an effect size in that it reflects standardized mean differences between groups, i.e., there was a small but reliable reduction in VAS symptoms in the treatment group compared to the control group. Note, that the test-retest reliability of the THQ (*r* = .922) can be considered as satisfactory, hence no a latent change score analysis was performed for this primary outcome measure.

#### Subjective evaluation of treatment by participants

##### Music enjoyment

A mixed model ANOVA with the within subjects factor *Session* (pre vs. post) and the between subjects factor *Group* (treatment vs. placebo) showed a significant main effect for the factor *Session*, *F* (1, 81) = 59.37, *p* < .000, *η*_*p*_^*2*^ = .42. Thus, music enjoyment during the last 4 weeks of the training was significantly lower than general enjoyment of music before the training (pre: *M* VAS music enjoy ratings = 82.98, *SD* = 16.19; post: *M* VAS music enjoy ratings = 63.86, *SD* = 21.18). However, there was no interaction between groups, *F* (1, 81) = 1.51, *p* = .222.

##### Music listening times

Due to technical problems, only the data of 78 participants could be analyzed. On average, participants listened to the provided music on 71.56 days (*SD* = 16.36) for 115.29 min per day (*SD* = 16.01), which did not differ between groups (days listened: *t* (76) = −0.52, *p* = .604; minutes per day listened: *t* (76) = 0.43, *p* = .669).

##### Subjective ratings of training benefit and judgement of group allocation

Table 9 depicts the subjective ratings of training benefit of the CGI scale. The judgments of group allocation by the participants are shown in Table 10.

**Table 9:** Subjective ratings of training benefit

	Treatment	Placebo	Total
my tinnitus improved very much	1	0	1
my tinnitus improved a bit	9	5	14
my tinnitus did not change	16	30	46
my tinnitus worsened a bit	12	7	19
my tinnitus worsened very much	2	1	3

**Table 10:** Judgements of group allocation

	Treatment	Placebo	Total
Judged placebo	38	31	69
Judged treatment	7	12	19

### Harms

All participants were asked to report if they perceived any harm or side effects after music exposure. Of all participants who filled out a questionnaire asking for harms (*n* = 92), the main harms reported were (1) a consistently louder tinnitus (10.9 %), (2) the perception of an additional sound (8.7 %), and (3) an increased awareness of the tinnitus sound (6.5 %). However, harms did not differ significantly between groups, *χ*^2^ (2) = 0.849, *p* = .654. In total, 29.4 % of the participants reported some form of harm, which is outlined in Table 11.

**Table 11:** Reported harms after tailor-made notched training (post measurements)

	Treatment	Placebo	Total
Number of participants with harms	12	15	27
Consistently louder	5	5	10
Additional sound	4	4	8
More aware of tinnitus sound	2	4	6
At times louder	2	2	4
Changes in sense of hearing	0	3	3
Tinnitus sound changes	0	3	3
Psychological stress	1	2	3
Other bodily changes	0	2	2

## Discussion

This was the first randomized double blind clinical trial on TMNMT. Overall, we did not find the hypothesized reduction of tinnitus related distress and tinnitus perception in the primary outcome measures, while in secondary outcome measures a reduction of tinnitus loudness in the treatment group was evident.

For the predefined primary outcome measures, TMNMT was not superior to a placebo treatment directly after training cessation. Tinnitus distress scores and scores for the subjective tinnitus perception remained at the same level for the treatment and the placebo group. The results of the intention-to-treat analysis did not differ indicating that dropouts did not influence the results of the primary outcome measures. Despite our previous reasoning, more time may be necessary to create and stabilize more pronounced effects of TMNMT on tonal tinnitus. In the study of Okamoto et al. [17], TMNMT affected tinnitus loudness after 6 months but it was shown to be superior to the placebo condition after 12 months. It is plausible that the effect after three month was too small to be detected in behavioral measures directly after training cessation.

However, more recent studies suggest that short-term changes in neurophysiological brain activity elicited through TMNMT already begin to occur after three days [19, 20]. Brain plasticity effects are often first observed on a neural level and may only later manifest behaviorally [53]. Thus, TMNMT might need more time to influence the subjective tinnitus perception.

Considering our secondary outcome measures, effects differed between the placebo and the target group for the TQ only. Contrary to our hypothesis, the placebo group showed even lower scores in the TQ after the training when compared to the treatment group. However, the effect did not reach significance when the groups were analyzed separately. One explanation for the contradicting effect in the TQ scores might be regression to the mean, as the two groups differed slightly (but not significantly) at the baseline measurement: The placebo group had higher scores in the TQ than the treatment group and therefore had a higher probability to show a reduction at the second measurement point (post training). Note, that for the TQ, the interaction was no longer visible in the follow-up measurement. It remains unclear, whether this small negative effect was due to influence of TMNMT or a regression to the mean. The effect of the reduction in tinnitus awareness in both groups from pre to post measurement, and the further decrease in the follow-up measurement, can be explained by elevated tinnitus awareness at the start of the trial. As participants expected changes in their tinnitus, they might have monitored their tinnitus more carefully prior to and during the training. The reduction towards the end of the trial can be seen as a returning to a normal level.

In the follow-up analysis we found a significant reduction of tinnitus loudness in the treatment group, while the scores in the placebo group reversed to baseline. According to Adamchic & Langguth (2012) the change in the loudness ratings of 6.22 points are not clinically relevant as changes of visual analog scales need to be greater than 10 points to be considered as an MCID. However, despite the relatively small effect, this result proved to be lasting. This can be explained by the fact that one month after cessation of the training, any placebo effect had faded, while the effect of TMNMT was persisting. Furthermore, this result underlines the above statement that the behavioral effects of TMNMT need more time than 3 months to evolve and stabilize. Given the reliability calculations within our study, we added the calculation of an SEM to our analysis. The calculation of an SEM resulted in a rather small effect size, but hints towards a positive effect of TMNMT as compared to placebo treatment in the post measurements for the mean VAS ratings. The result of this analysis has to be interpreted with care, as the analysis was not prevised in our protocol and further research is needed to confirm this preliminary data. Taken together with the reduction of tinnitus loudness, which was found in the follow-up measurement, we would conclude that 3 months of TMNMT had a relatively small, but persistent influence on the subjective tinnitus perception.

A considerable number of participants reported the experience of adverse effects during the duration of the trial. We do not know if the number of reported adverse effects is comparable or equal to previous studies, as none of the previous studies concerning TMNMT has asked for adverse effects with an open question item [17, 18, 54]. However, those adverse effects appeared to be unspecific and not caused by TMNMT, as they were evenly distributed between the two groups. Therefore, the adverse effects that participants experienced could be interpreted as general effects of attention towards the tinnitus due the participation in the study. Furthermore the adverse effects might be general symptoms associated with tinnitus that would have appeared even without participating in the study. A monitoring group would have given more insight to this.

How clear is the evidence of 3 months TMNMT as treatment against tinnitus? The results of the current study point in different directions: Directly after the training, we observed no significantly positive effect with the proposed analysis. Even the reverse was true, as for one of the secondary measurements of tinnitus distress, a negative development was found in the treatment group when compared to the placebo group. This small effect, however, was not observable 1 month after training cessation. On the other hand, a reduction in tinnitus loudness, awareness, handicap and annoyance due to TMNMT was found with more explorative analysis. This hint could be strengthened by the observation of a reduction of tinnitus loudness in the TMNMT group 1 month after training cessation. Taken together, our results display a positive effect of three months of TMNMT on the subjective tinnitus perception, especially concerning tinnitus loudness. These results are in line with the previous placebo controlled study of Okamoto et al. [17], which demonstrated a positive effect of TMNMT on the VAS loudness scale as well. Other studies of TMNMT had different designs and training durations and used varying outcome measures making it difficult to compare the results. Okamoto et al. [17] used VAS and calculated relative change values, Teismann et al. [13] used the THQ, the TQ and VAS scales, but did not find effects for all measures at all time points. Teismann et al. [54] found effects for the THQ but not for the VAS. Stein et al. [19] as well as Stein et al. [20] found effects in the VAS. So far, these studies have been performed using smaller samples and most of them did not have a placebo group to control the effects. Considering the placebo controlled studies only, consistent effects of TMNMT are observable for subjectively perceived tinnitus loudness. Longer time of at least twelve months [17] may be necessary to stabilize the positive effects of TMNMT on tonal tinnitus.

Within the research on sound therapies, clinical trials focusing on the effectiveness of a specific sound treatment without any other combined strategy are rather seldom [5]. Hence, this study offers valuable insights into the effectiveness of a specific sound therapy as a stand-alone treatment against tinnitus. Analogously to previous studies on sound therapies, the significant effects we have obtained after only 3 months of training are rather small, however still persisting in the follow-up measurements after 1 month. Therefore, it could be a goal to further improve the effectiveness of TMNMT and to integrate it in a more holistic approach of tinnitus treatment.

### Limitations

Tinnitus-related distress was generally low in our sample, even before the training. High levels of tinnitus distress are often accompanied by mental disorders [55]. Through our exclusion criterion regarding mental disorders like depression, we might have likewise excluded participants with higher distress levels. This could have led to a floor effect, where an improvement was more difficult to detect. On the other hand, mental disorders were an exclusion criterion in all previous studies investigating TMNMT. Therefore, it still remains unclear whether TMNMT would be suitable for participants that are highly distressed by their tinnitus. Importantly, hearing loss, age and tinnitus frequency criteria are factors, which differed from previous TMNM studies. Yet, covariate analyses with these factors did not show any influence on the results. Therefore, we conclude that differences between this study and previous studies regarding those variables are negligible.

Another limitation is the psychometric quality of our outcome measures. As mentioned above, not all outcome measures were specifically developed to measure change in clinical trials. A relatively new and well-described questionnaire is the Tinnitus Functional Index (TFI) [56]. This questionnaire differentiates between different domains of tinnitus distress and the authors provide a grading system and a MCID score to be used for clinical interpretation [27]. Therefore, it appears to be a measure that should be considered as an outcome measure in future clinical trials for tinnitus. However, when our clinical trial was started, the evaluation of the TFI was not completed nor was a German version available and evaluated.

## Conclusions

This is the first study on TMNMT that was planned and conducted following the CONSORT statement standards for clinical trials. The current work is one more step towards a final evaluation of TMNMT. Already after 3 months the effect of training with TMNM is observable in the most direct rating of tinnitus perception – the tinnitus loudness, while more global measures of tinnitus distress do not show relevant changes.

## Abbreviations

TMNMT: tailor-made notched music training
CONSORT: consolidated standards of reporting trials
VAS: visual analogue scales
TRT: tinnitus retraining therapy
THQ: Tinnitus Handicap Questionnaire
TQ: Tinnitus Questionnaire
THI: Tinnitus Handicap Inventory
ENT: Ear, Nose and Throat
TMNM: tailor-made notched music
ADS-L: Allgemeine Depressionsskala – Langfassung (Questionnaire measuring Depression)
SCL-90-R: symptom checklist for general psychological distress
MCID: minimally clinically important difference
GüF: Geräuschüberempfindlickeits-Fragebogen (Questionnaire measuring hyperacusis)
CGI: Clinical Global Impression
ANOVA: analysis of variance
ANCOVA: analysis of covariance
SEM: structural equation model.
